# Facial protection of hats and caps against ultraviolet radiation: experimental study in Ica, Peru

**DOI:** 10.17843/rpmesp.2026.432.15080

**Published:** 2026-06-04

**Authors:** Ubaldo Miranda-Soberón, Stefanie Guerrero-Jhong, Yovanny Buleje-Mendoza, Hugo Arroyo-Hernández

**Affiliations:** 1 Facultad de Medicina, Universidad Nacional San Luis Gonzaga, Ica, Peru.; 2 Semillero CLIMA, Facultad de Medicina, Universidad Nacional San Luis Gonzaga, Ica, Peru.; 3 Facultad de Ciencias, Universidad Nacional San Luis Gonzaga, Ica, Peru.; 4 Universidad de Huánuco, Huánuco, Peru.

Dear Editor. Ultraviolet (UV) radiation can cause skin cancer, mainly in exposed areas like the face ^1^^,^^2^. The harmful effects depend on exposure time and geography ^3^, so it is essential to use chemical protection, such as sunscreen, and mechanical protection, such as hats, glasses, and appropriate clothing, to mitigate these risks ^2^^,^^4^.

This research aimed to evaluate the effectiveness of head coverings used for facial protection against UV radiation. Twenty units were analyzed, classified into three groups: caps (n=4, with front visors), walking hats (n=8, with medium brims), and peasant hats (n=8, with wide brims), the latter commonly used in agricultural work.

The garments were placed on a mannequin made of rigid polymeric material, with a matte, light-colored surface, and defined facial features (supplementary material). The model was positioned oriented northward at the geographical coordinates 14°05´25.80”S, 75°45´05.05”W, corresponding to an open area in the district of Ica, Peru. This region is characterized by an arid geography with low cloud cover and a dry desert climate, conditions that favor high solar incidence. Measurements were performed between February 2 and 21, 2024.

The width of the garment wings (cm), internal and external diameter (cm), as well as their shape and color were measured. UV radiation was recorded in milliwatts mW/cm*²* at five anatomical sites (ears, chin, lips, nose, and neck) on both the right and left sides. To capture the variability of protection according to the solar angle, a hourly exposure cycle from 09:00 to 15:00 hours was completed. Measurements were systematically distributed under clear sky conditions, performing paired readings (with and without garment). Protective efficacy was defined as the percentage of UV radiation blocked on a scale of 0 to 100%, calculated with the following equation:


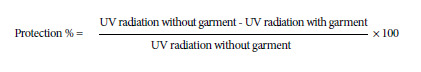


UV radiation was measured with a portable radiometer model PCE-UV/34 (PCE Instruments), with a measurement range of 2 to 20 mW/cm² and an external sensor sensitive to wavelengths between 290 and 390 nm, covering the UVA and UVB spectrum. The setup process included a zero adjustment to eliminate dark currents in the absence of radiation. Subsequently, a field validation was performed through simultaneous measurements with a reference radiometer from the Servicio Nacional de Meteorologia e Hidrologia del Peru (SENAMHI). Both devices were placed at the same point and orientation, recording readings at different times of the day to cover a range of solar intensities. The data obtained showed a correlation coefficient of 0.83, which indicates a very good agreement for the identification of temporal variations and relative comparisons. No correction factor was applied given that the measurements were consistent and remained within the ranges of physical plausibility. Finally, all readings were collected by a single trained operator to minimize technical variability and ensure the absence of invalid records.

UV protection percentages were aggregated and expressed as medians and interquartile range (IQR). The unit of analysis was each head protection garment. For each garment, the percentage of protection per anatomical site was initially calculated, averaging bilateral measurements when applicable; subsequently, a global median was estimated as an indicator of facial protection. Regarding the time of day, the median global protection percentage was calculated for each time slot. Due to the non-normal distribution of the data and the sample size, comparisons among the three types of hats were performed using the Kruskal-Wallis test. Given significant overall differences (p<0.05), Dunn's test was applied for post hoc multiple comparisons. The analyses were performed using InfoStat software.

55% of the garments were circular and 35% were light-colored. The brim varied between 6 and 16 cm. The major diameter between 26 and 48 cm and the minor between 18 and 48 cm. The peasant hat obtained a significantly higher median facial protection (83.1%) than the walking hat and the cap (p=0.001). The ears were the anatomical site with the highest overall protection, with a median of 92.9% where peasant hats and walking hats showed greater protection than caps, but without significant differences. In contrast, for the nose, lips, and neck, peasant hats demonstrated greater protection with a significant difference when compared to other models. The protection of peasant hats was also superior during peak radiation hours (11:00 to 13:00 h) with a significant difference when compared to walking hats and caps (table 1).

**Table 1:** Solar protection level achieved by three types of head coverings according to measurement sites and times of day.

		Total (N=20)	Type of headwear			
			Cap (n=4)	Walking hat (n=8)	Farmer's hat (n=8)	p-value*
		Median % overall protection (IQR)	Median of the % protection (IQR)	Median of the % protection (IQR)	Median of the % protection (IQR)	
Facial protection		70.3 (55.0 - 79.8)	44.5 (32.1 - 54.5)^a^	62.0 (55.0 - 72.6)^a^	83.1 (76.6 - 87.3)^b^	0.001
Measurement site						
	Ear	92.9 (77.8 - 94.3)	74.3 (39.2 - 84.1)	92.8 (82.2 - 93.6)	93.9 (92.7 - 94.9)	0.116
	Nose	88.1 (74.8 - 92.7)	67.0 (51.1 - 74.8)^a^	82.9 (77.7 - 88.9)^a^	92.9 (91.9 - 94.4)^b^	0.002
	Lips	66.0 (49.0 - 85.2)	30.0 (25.4 - 42.3)^a^	55.5 (50.6 - 69.4)^a^	87.6 (80.8 - 89.2)^b^	0.001
	Neck	54.3 (40.8 - 71.6)	38.7 (23.9 - 47.1)^a^	47.3 (35.8 - 58.5)^a^	75.6 (68.5 - 83.7)^b^	0.007
	Chin	49.6 (29.9 - 63.3)	19.5 (17.3 - 24.3)^a^	32.7 (31.7 -50.8)^b^	71.8 (57.8 - 78.4)^c^	0.001
Time of day						
	9	49.2 (31.2 - 71.3)	28.9 (17.1 - 35.1)^a^	40.5 (29.7 - 53.5)^a^	73.9 (65.9 - 77.1)^b^	0.001
	10	64.1 (46.5 - 78.4)	37.6 (20.4 - 46.8)^a^	55.0 (43.9 - 67.5)^a^	80.3 (76.2 - 86.2)^b^	0.001
	11	79.1 (52.4 - 89.5)	48.2 (33.9 - 54.4)^a^	72.5 (52.4 - 80.1)^a^	91.5 (88.1 - 92.6)^b^	0.001
	12	79.2 (64.9 - 89.0)	52.0 (35.9 - 66.6)^a^	75.6 (65.8 - 83.0)^a^	89.9 (88.3 - 91.5)^b^	0.003
	13	75.8 (67.4 - 90.0)	54.6 (47.3 - 66.8)^a^	73.7 (71.2 - 78.9)^a^	90.8 (83.0 - 92.9)^b^	0.006
	14	70.4 (51.2 - 75.4)	47.8 (43.3 - 59.6)^a^	62.1 (50.4 - 70.7)^a^	77.5 (72.6 - 88.4)^b^	0.004
	15	57.4 (47.1 - 63.7)	49.6 (36.9 - 59.4)^a^	53.8 (40.2 - 58.9)^a^	67.0 (54.9 - 73.0)^b^	0.047

RIC: Interquartile range, * Kruskal-Wallis test.

a, b, c : groups with different letters in the same row indicate significant differences (p<0.05) according to Dunn's test.

Measurement site: anatomical points where the average percentage of protection of both sides of the face was calculated.

Protection was defined as the percentage of UV radiation blocked on a scale of 0 to 100%.

Facial protection: defined as the global median of protection percentages at all measurement sites.

Time of day: represents the median of the overall protection percentage calculated within the measurement time slots.

These results coincide with other reports ^3^^,^^5^^,^^6^ where caps showed limited protection compared to wide-brimmed hats, in most facial sites. However, caps are the most used design ^7^. Likewise, it should be considered that effectiveness not only depends on the type of hat, but also on the time of use, as well as social norms, costs, and other determinants.

An important limitation of the study is the use of a static experimental model to represent a dynamic reality. The experiment was conducted using an immobile mannequin, while in real conditions people move and turn their heads, which influences exposure to UV radiation. Additionally, weather conditions vary throughout the day as well as during the year. Consequently, the values probably overestimate protection under use conditions. Likewise, the study evaluated garments selected by convenience and is not representative of the universe available in Peru. Finally, the study was conducted exclusively in an area with particular geographical and solar radiation characteristics, which restricts the generalization of the findings.

In conclusion, wide-brimmed hats such as sun hats and, to a lesser extent, fedora hats protect against UV radiation more than caps. It should be noted that hats will never protect 100%, so their use must be complemented with other preventive measures. Considering the increase in solar radiation ^8^, implementing protection strategies, knowing their efficacy and safety, is essential to prevent skin damage.
