# Supplementary material for: Facial protection of hats and caps against ultraviolet radiation: experimental study in Ica, Peru
Source: Rev Peru Med Exp Salud Publica. 2026 Jun 4;43(2):281–3. doi: 10.17843/rpmesp.2026.432.15080 (PMC13456930; doi:10.17843/rpmesp.2026.432.15080)

Material Suplementario.

Se muestran fotografías de algunas de las prendas de cobertura para la cabeza utilizadas en el estudio. Las prendas fueron clasificadas en tres grupos: a) gorras (con visera frontal), b) sombreros de paseo (con ala intermedia) y c) sombreros campesinos (de ala ancha).
Las prendas fueron colocadas sobre un maniquí de material polimérico rígido, de superficie mate y color claro, con rasgos faciales. El maniquí se posicionó orientado al norte en las coordenadas geográficas 14°05´25.80”S, 75°45´05.05”W, correspondiente a un área abierta en el distrito de Ica, Perú.

a) Gorras

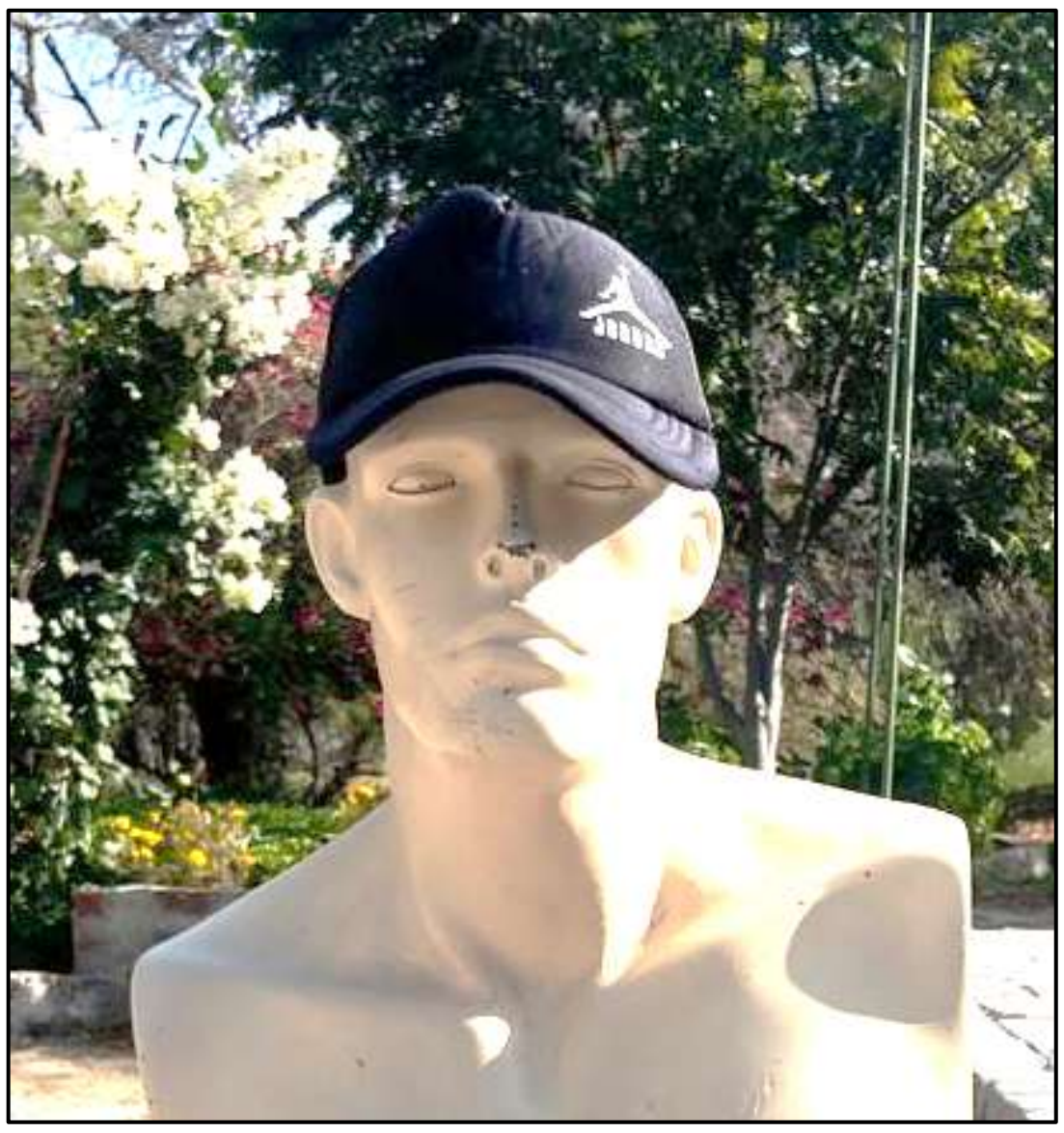

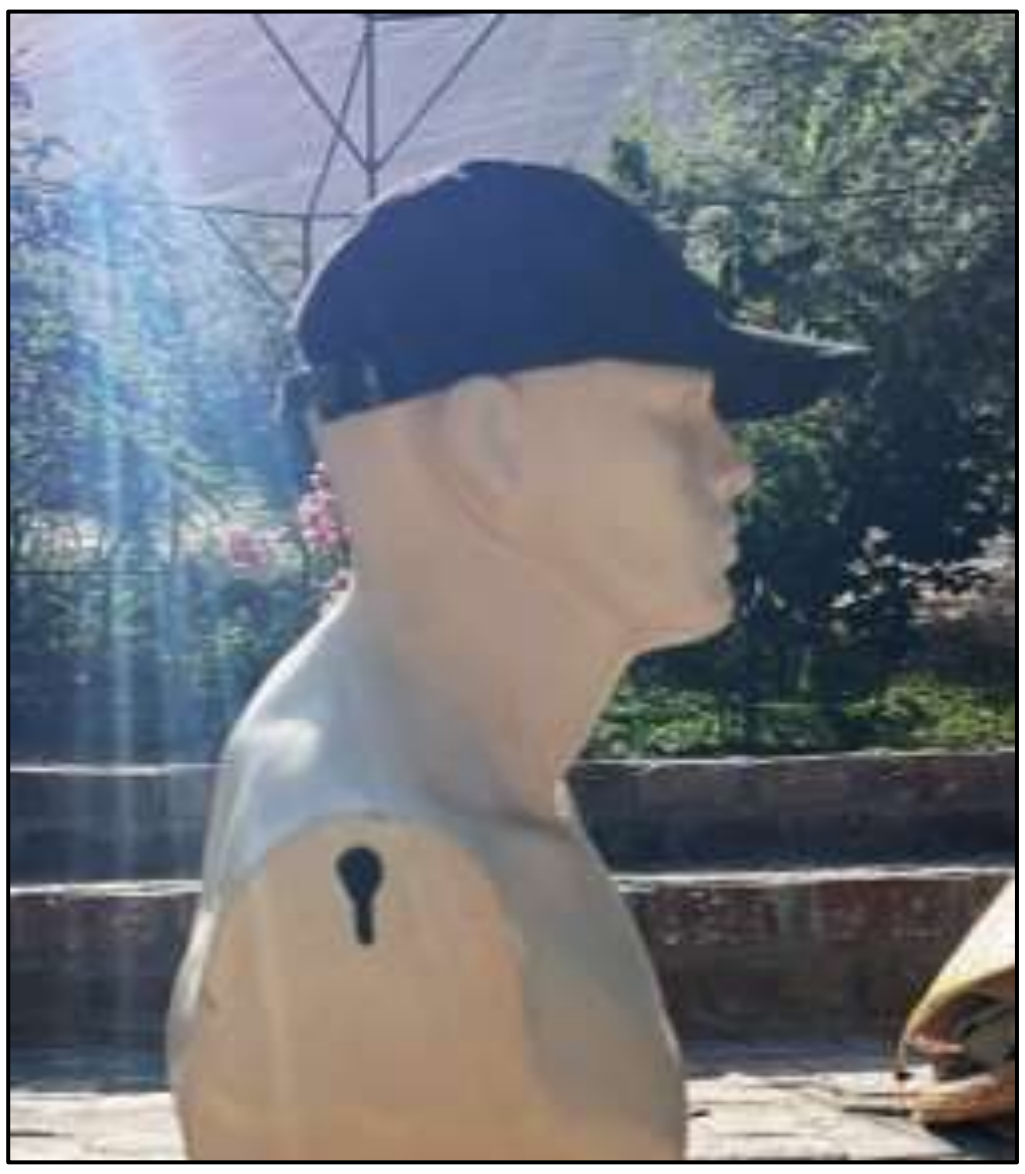

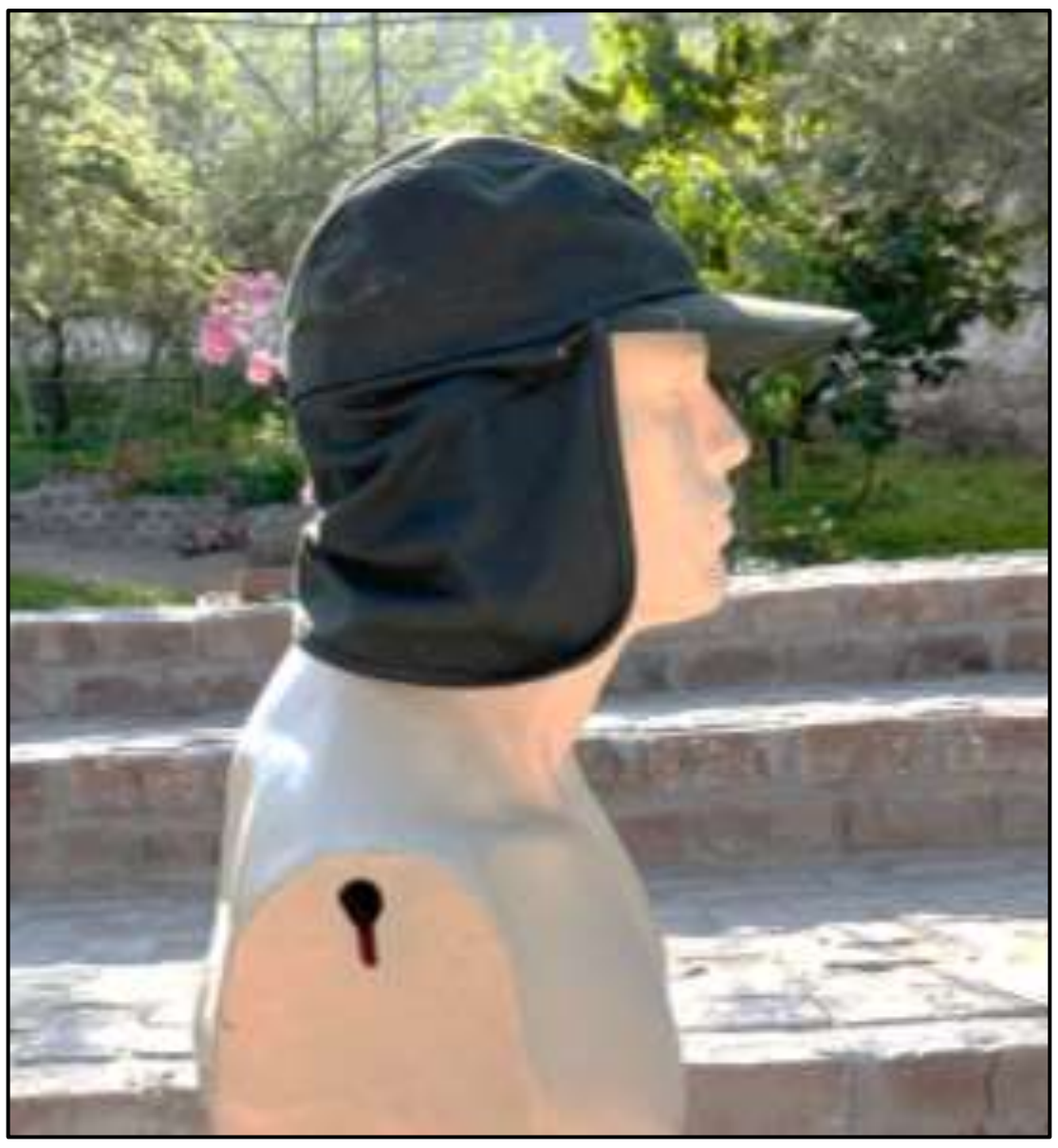

b) Sombreros de paseo.

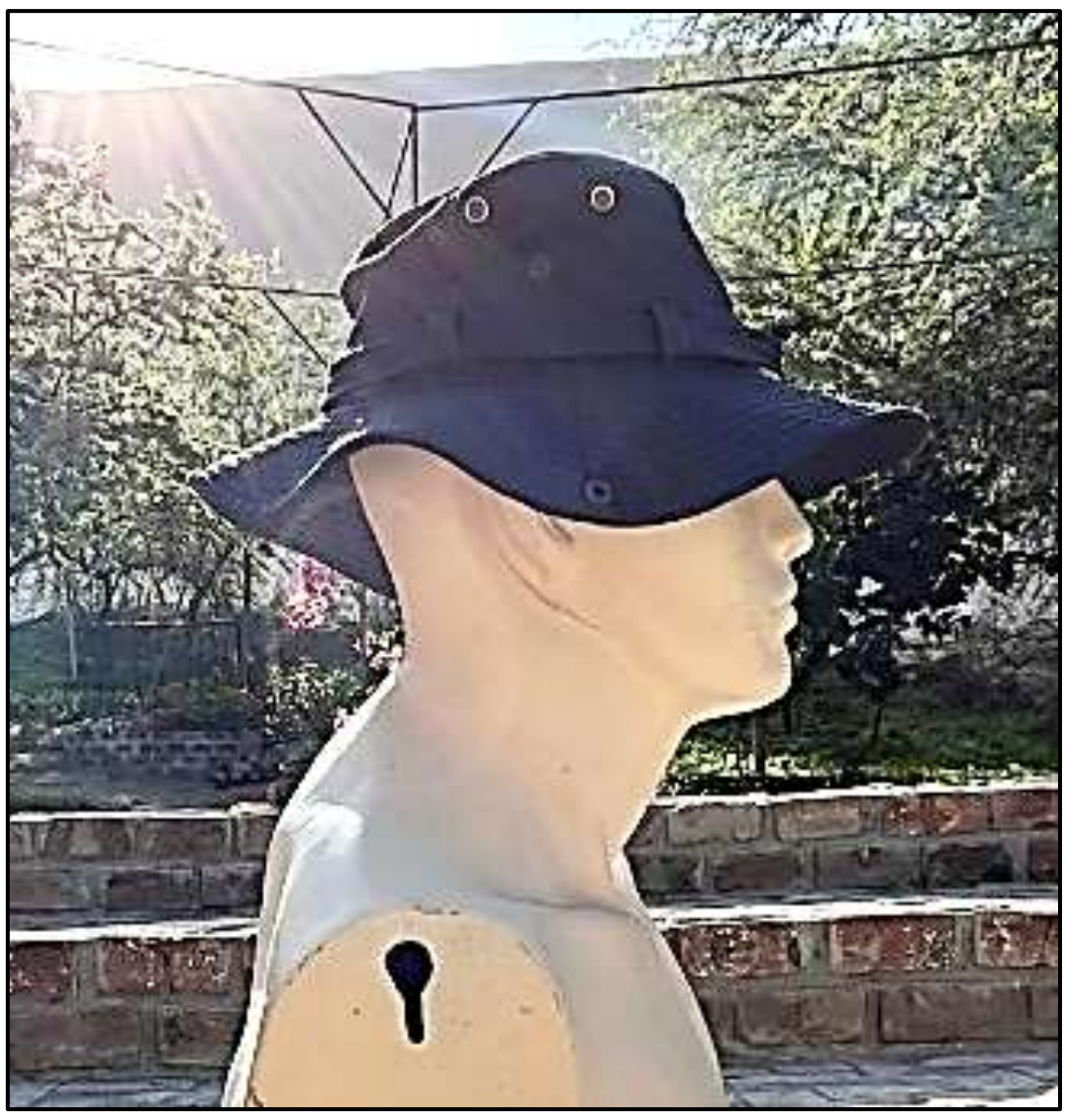

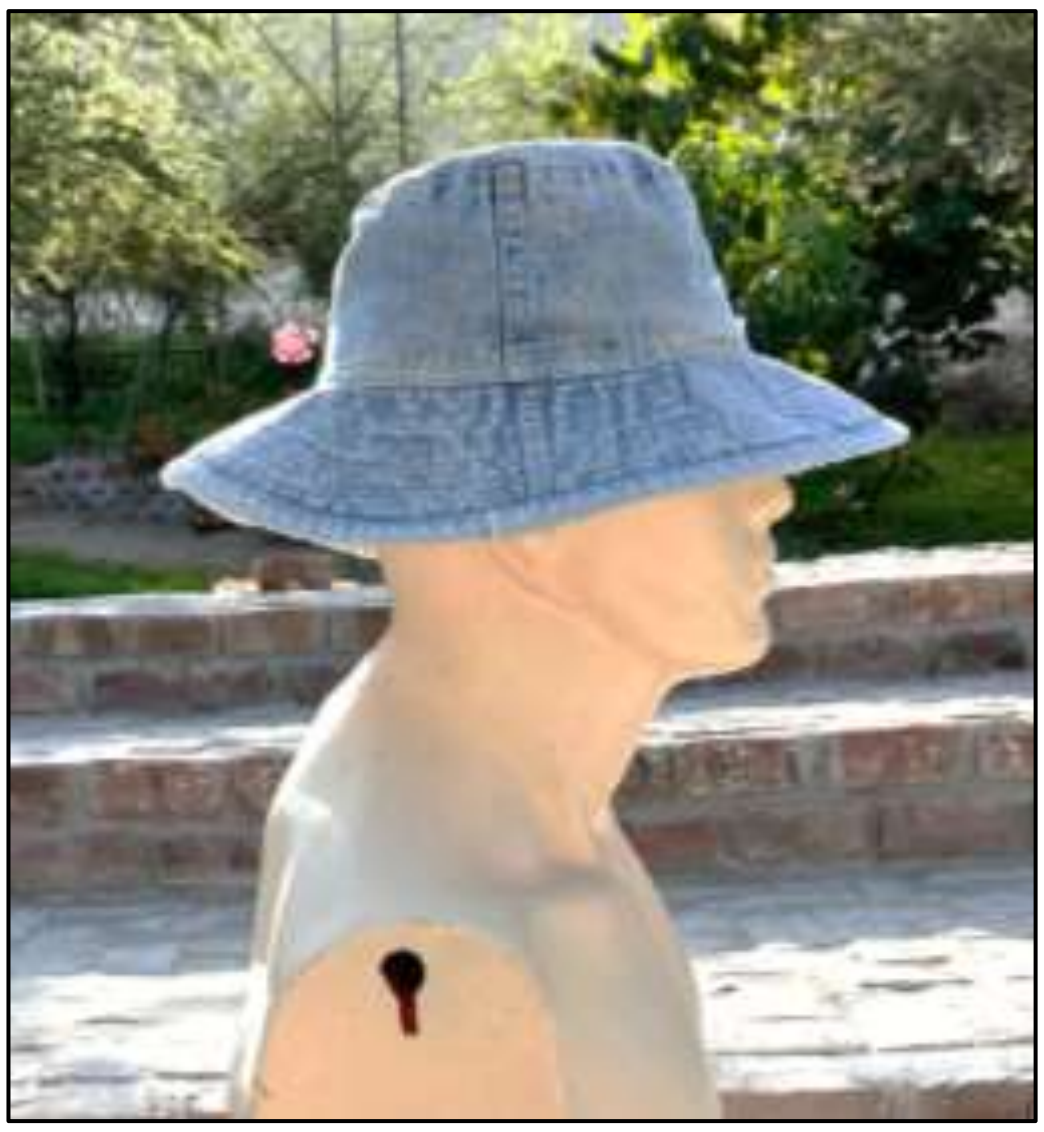

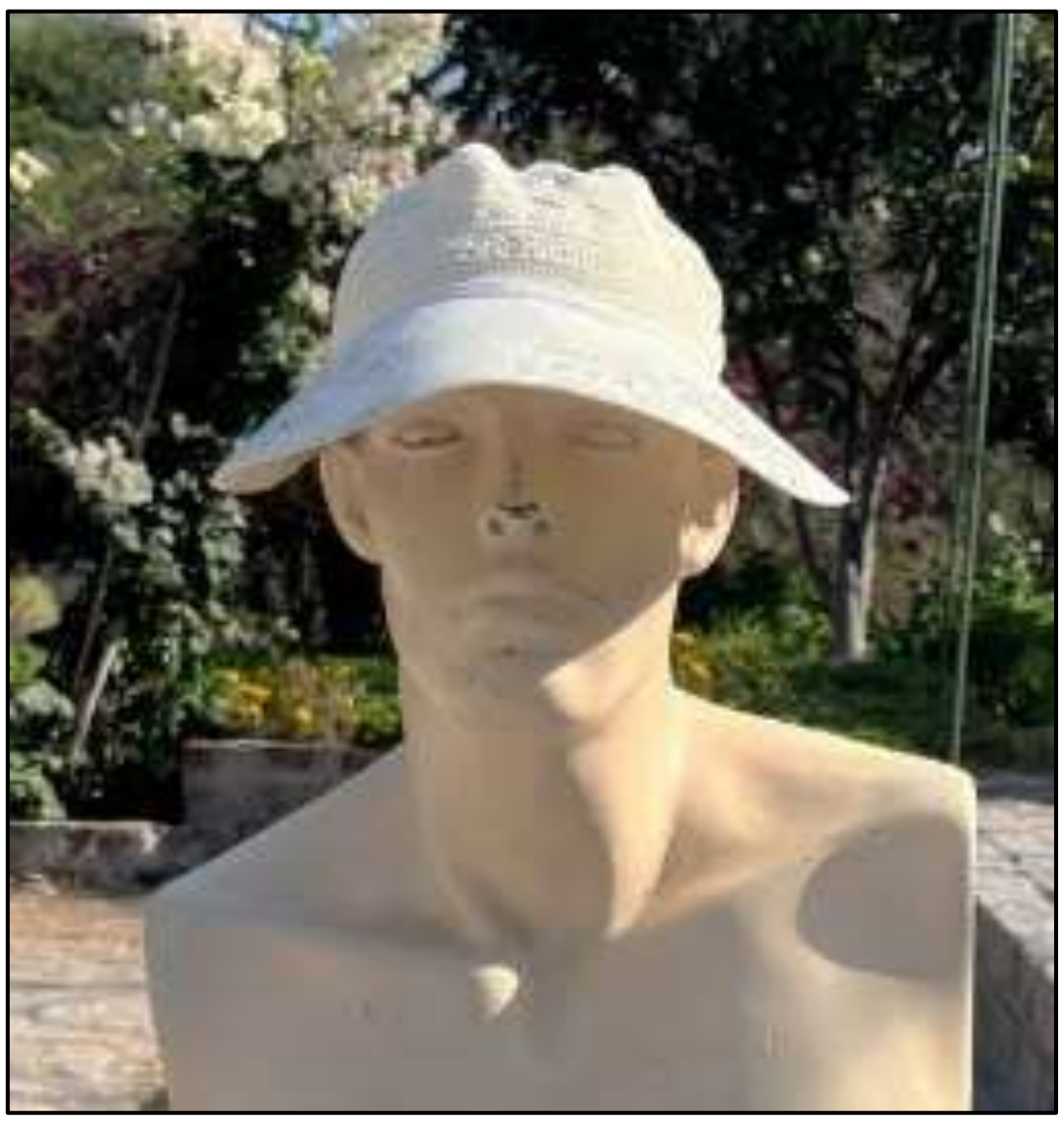

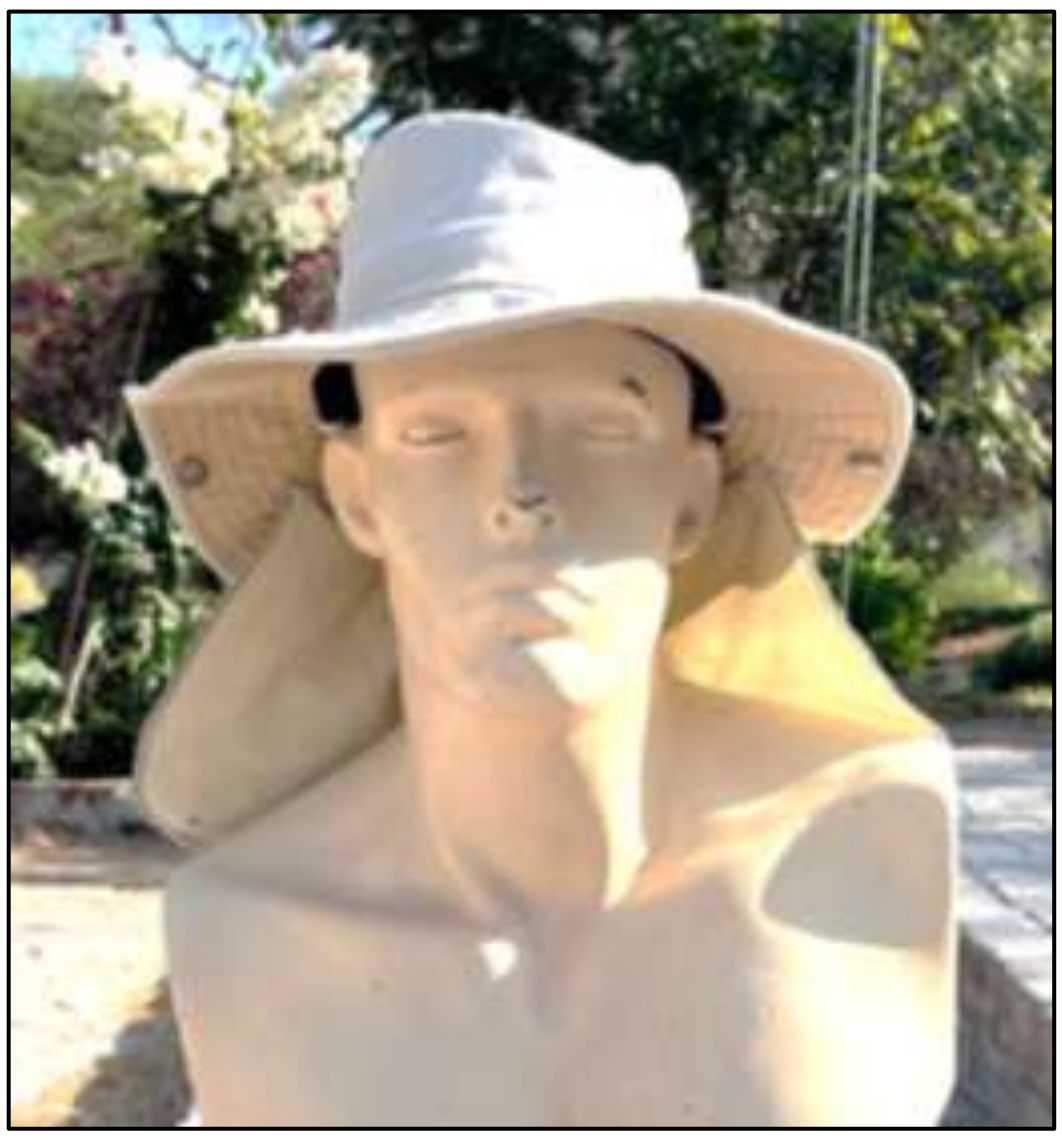

c) Sombreros campesinos.

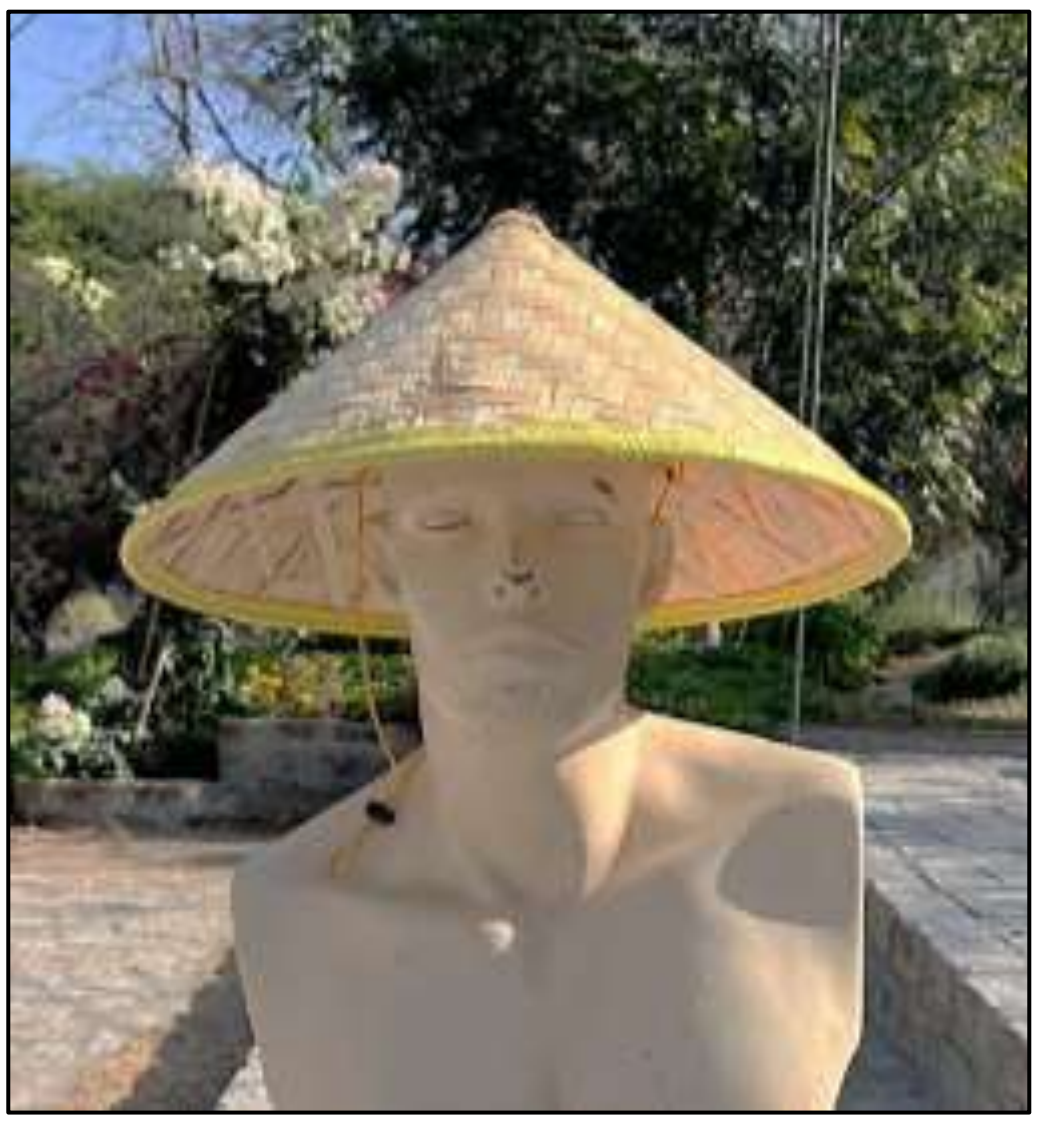

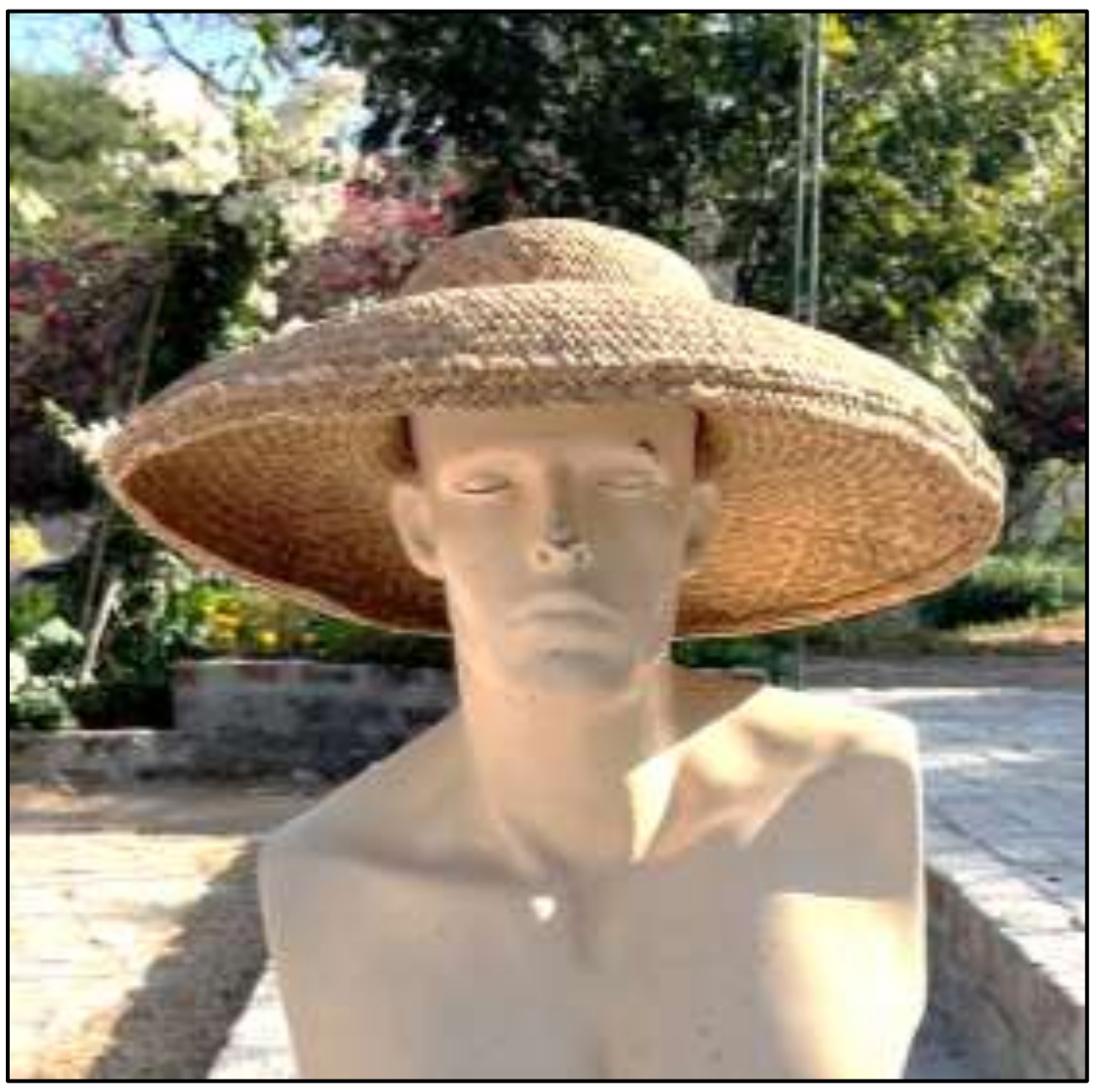

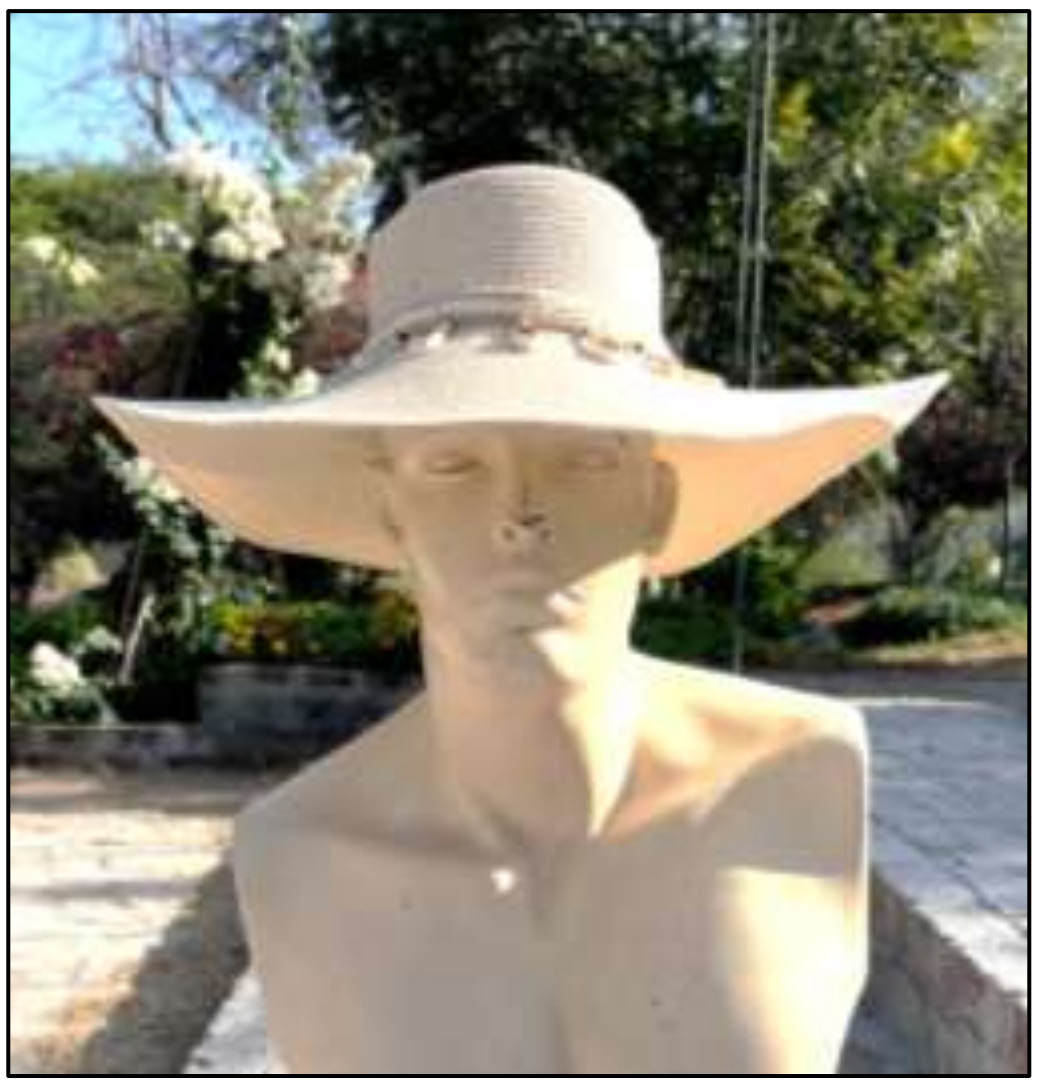

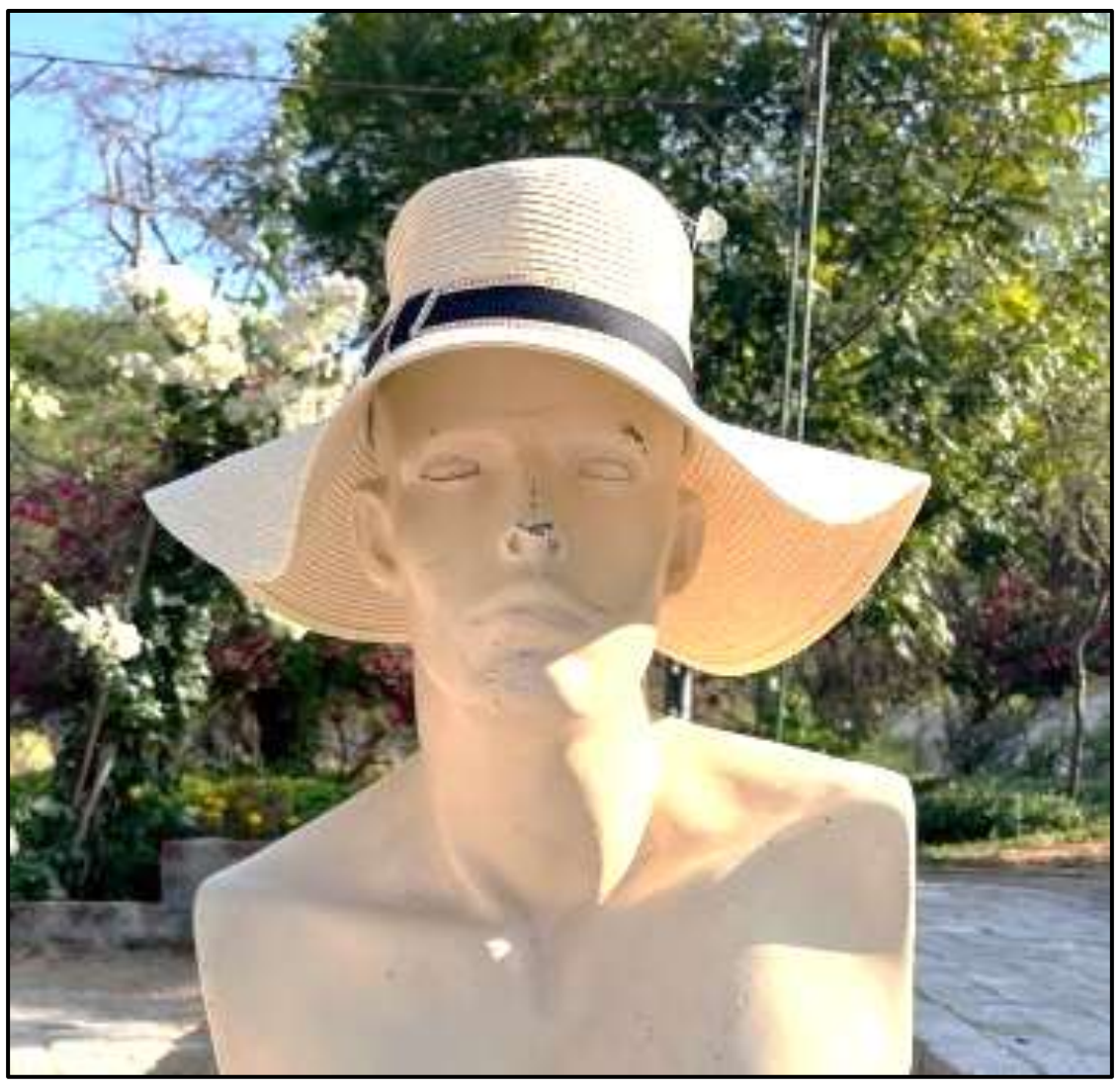

Supplement: Supplementary file 2 [file rpmesp-43-02-15080-s001.pdf]
