# Supplementary material for: Facial protection of hats and caps against ultraviolet radiation: experimental study in Ica, Peru
Source: Rev Peru Med Exp Salud Publica. 2026 Jun 4;43(2):281–3. doi: 10.17843/rpmesp.2026.432.15080 (PMC13456930; doi:10.17843/rpmesp.2026.432.15080)
Supplement: Translation

# Translation: Protección facial de sombreros y gorras frente a la radiación ultravioleta: estudio experimental en Ica, Perú

**Authors:** Ubaldo Miranda-Soberón, Stefanie Guerrero-Jhong, Yovanny Buleje-Mendoza, Hugo Arroyo-Hernández

**Affiliations:** 1 Facultad de Medicina, Universidad Nacional San Luis Gonzaga, Ica, Perú.; 2 Semillero CLIMA, Facultad de Medicina, Universidad Nacional San Luis Gonzaga, Ica, Perú.; 3 Facultad de Ciencias, Universidad Nacional San Luis Gonzaga, Ica, Perú.; 4 Universidad de Huanuco, Huanuco, Perú.

Sr. Editor. La radiación ultravioleta (UV) pueden causar cáncer de piel, principalmente en zonas expuestas como la cara ^1^^,^^2^. Los efectos perjudiciales dependen del tiempo de exposición y de la geografía ^3^, por lo que es esencial usar protección química, como el bloqueador solar, y mecánica, como sombreros, lentes y ropa adecuada, para mitigar estos riesgos ^2^^,^^4^.

Esta investigación tuvo como objetivo evaluar la eficacia de las prendas de cobertura para la cabeza utilizadas para protección facial frente a la radiación UV. Se analizaron 20 unidades, clasificadas en tres grupos: gorras (n=4, con visera frontal), sombreros de paseo (n=8, de ala intermedia) y sombreros campesinos (n=8, de ala ancha), estos últimos utilizados habitualmente en labores agrícolas.

Las prendas fueron colocadas sobre un maniquí de material polimérico rígido, de superficie mate y color claro, con rasgos faciales definidos (material suplementario). El modelo se posicionó orientado al norte en las coordenadas geográficas 14°05´25.80”S, 75°45´05.05”W, correspondientes a un área abierta en el distrito de Ica, Perú. Esta región se caracteriza por una geografía árida con escasa nubosidad y un clima desértico seco, condiciones que favorecen una alta incidencia solar. Las mediciones se realizaron entre el 2 y el 21 de febrero del 2024.

Se midió el ancho del ala (cm) de las prendas, diámetro interno y externo (cm), además de su forma y color. La radiación UV se registró en miliwatios mW/cm*²* en cinco sitios anatómicos (orejas, mentón, labios, nariz y cuello) tanto del lado derecho como izquierdo. Para capturar la variabilidad de la protección según el ángulo solar, se completó un ciclo horario de exposición de 09:00 a 15:00 horas. Las mediciones se distribuyeron de forma sistemática bajo condiciones de cielo despejado, realizando lecturas pareadas (con y sin prenda). La eficacia protectora se definió como el porcentaje de radiación UV bloqueada en una escala de 0 a 100%, calculado con la siguiente ecuación:



La radiación UV se midió con un radiómetro portátil modelo PCE-UV/34 (PCE Instruments), con un rango de medición de 2 a 20 mW/cm² y un sensor externo sensible a longitudes de onda entre 290 y 390 nm, cubriendo el espectro UVA y UVB. El proceso de puesta a punto incluyó un ajuste a cero, para eliminar corrientes de oscuridad en ausencia de radiación. Posteriormente, se realizó una validación de campo mediante mediciones simultaneas con un radiómetro de referencia del Servicio Nacional de Meteorología e Hidrología del Perú (SENAMHI). Ambos equipos se ubicaron en el mismo punto y orientación, registrando lecturas en distintos momentos del día para cubrir un rango de intensidades solares. Los datos obtenidos mostraron un coeficiente de correlación de 0,83, lo que indica una concordancia muy buena para la identificación de variaciones temporales y comparaciones relativas. No se aplicó un factor de corrección dado que las mediciones fueron consistentes y se mantuvieron dentro de los rangos de plausibilidad física. Finalmente, todas las lecturas fueron recolectadas por un único operador entrenado para minimizar la variabilidad técnica y garantizar la ausencia de registros inválidos.

Los porcentajes de protección UV fueron agregados y expresados como medianas y rango intercuartílico (RIC). La unidad de análisis fue cada prenda de protección para la cabeza. Para cada prenda, se calculó inicialmente el porcentaje de protección por sitio anatómico, promediando las mediciones bilaterales cuando correspondía; posteriormente, se estimó una mediana global como indicador de protección facial. En relación con la hora del día, se calculó la mediana del porcentaje de protección global para cada franja horaria. Debido a la distribución no normal de los datos y el tamaño muestral, las comparaciones entre los tres tipos de sombrero se realizaron mediante la prueba de Kruskal-Wallis. Ante diferencias globales significativas (p<0,05), se aplicó la prueba de Dunn para comparaciones múltiples post hoc. Los análisis se realizaron en el software InfoStat.

El 55% de las prendas fueron circulares y el 35% de colores claros. El ala varió entre 6 y 16 cm. El diámetro mayor entre 26 y 48 cm y el menor entre 18 y 48 cm. El sombrero campesino obtuvo una mediana de protección facial significativamente mayor (83,1%) que el sombrero de paseo y la gorra (p=0,001). Las orejas fueron el sitio anatómico con mayor protección general, con una mediana del 92,9% donde los sombreros campesinos y de paseo presentaron una mayor protección que las gorras, pero sin diferencias significativas. Por el contrario, en la nariz, los labios y el cuello, los sombreros campesinos demostraron una mayor protección con diferencia significativa al compararlo con otros modelos. La protección de los sombreros campesinos fue además superior durante las horas de máxima radiación (11:00 a 13:00 h) con diferencia significativa al compararlo con los sombreros de paseo y las gorras (tabla 1).

**Tabla 1:** Nivel de protección solar alcanzado por tres tipos de prendas de cobertura para la cabeza según sitios de medición y horas del día.

		Total (N=20)	Tipo de prendas para la cabeza			
			Gorra (n=4)	Sombrero de paseo (n=8)	Sombrero campesino (n=8)	Valor de p*
		Mediana del % protección general (RIC)	Mediana del % de protección (RIC)	Mediana del % de protección (RIC)	Mediana del % de protección (RIC)	
Protección facial		70,3 (55,0 - 79,8)	44,5 (32,1 - 54,5)^a^	62,0 (55,0 - 72,6)^a^	83,1 (76,6 - 87,3)^b^	0,001
Sitio de medición						
	Oreja	92,9 (77,8 - 94,3)	74,3 (39,2 - 84,1)	92.8 (82,2 - 93,6)	93,9 (92,7 - 94,9)	0,116
	Nariz	88,1 (74,8 - 92,7)	67,0 (51,1 - 74,8)^a^	82,9 (77,7 - 88,9)^a^	92,9 (91,9 - 94,4)^b^	0,002
	Labios	66,0 (49,0 - 85,2)	30,0 (25,4 - 42,3)^a^	55,5 (50,6 - 69,4)^a^	87,6 (80,8 - 89,2)^b^	0,001
	Cuello	54,3 (40,8 - 71,6)	38,7 (23,9 - 47,1)^a^	47,3 (35,8 - 58,5)^a^	75,6 (68,5 - 83,7)^b^	0,007
	Mentón	49,6 (29,9 - 63,3)	19,5 (17,3 - 24,3)^a^	32,7 (31, 7 -50,8)^b^	71,8 (57,8 - 78,4)^c^	0,001
Hora del día						
	9	49,2 (31,2 - 71,3)	28,9 (17,1 - 35,1)^a^	40,5 (29,7 - 53,5)^a^	73,9 (65,9 - 77,1)^b^	0,001
	10	64,1 (46,5 - 78,4)	37,6 (20,4 - 46,8)^a^	55,0 (43,9 - 67,5)^a^	80,3 (76,2 - 86,2)^b^	0,001
	11	79,1 (52,4 - 89,5)	48,2 (33,9 - 54,4)^a^	72,5 (52,4 - 80,1)^a^	91,5 (88,1 - 92,6)^b^	0,001
	12	79,2 (64,9 - 89,0)	52,0 (35,9 - 66,6)^a^	75,6 (65,8 - 83,0)^a^	89,9 (88,3 - 91,5)^b^	0,003
	13	75,8 (67,4 - 90,0)	54,6 (47,3 - 66,8)^a^	73,7 (71,2 - 78,9)^a^	90,8 (83,0 - 92,9)^b^	0,006
	14	70,4 (51,2 - 75,4)	47,8 (43,3 - 59,6)^a^	62,1 (50,4 - 70,7)^a^	77,5 (72,6 - 88,4)^b^	0,004
	15	57,4 (47,1 - 63,7)	49,6 (36,9 - 59,4)^a^	53,8 (40,2 - 58,9)^a^	67,0 (54,9 - 73,0)^b^	0,047

RIC: rango Intercuartílico, *prueba de Kruskal Wallis.

a, b, c : grupos con letras distintas en la misma fila indican diferencias significativas (p<0,05) según la prueba de Dunn.

Sitio de medición: puntos anatómicos donde se calculó el porcentaje promedio de protección de ambos lados del rostro.

La protección se definió como el porcentaje de radiación UV bloqueada en una escala de 0 a 100%.

Protección facial: definida como la mediana global de los porcentajes de protección en todos los sitios de medición.

Hora del día: representa la mediana del porcentaje de protección global calculado dentro de las franjas horarias de medición.

Estos resultados coinciden con otros reportes ^3^^,^^5^^,^^6^ donde las gorras mostraron una protección limitada comparada con los sombreros de ala ancha, en la mayoría de los sitios faciales. Sin embargo, las gorras son el diseño de mayor uso ^7^. Asimismo, se debe considerar que la efectividad no solo depende del tipo de sombrero, sino también del tiempo de uso, al igual que las normas sociales, costos, y otros determinantes.

Una limitación importante del estudio es el uso de un modelo experimental estático para representar una realidad dinámica. El experimento se realizó utilizando un maniquí inmóvil, mientras que en condiciones reales las personas se desplazan y giran la cabeza lo que influye en la exposición a la radiación UV. Adicionalmente, las condiciones meteorológicas varían a lo largo del día como durante el año. En consecuencia, los valores probablemente sobrestiman la protección bajo condiciones de uso. Asimismo, el estudio evaluó prendas seleccionados por conveniencia y no es representativo del universo disponible en Perú. Finalmente, el estudio se llevó a cabo exclusivamente en una zona con características geográficas y de radiación solar particulares, lo que restringe la generalización de los hallazgos.

En conclusión, los sombreros de ala ancha como los de tipo campesinos y, en menor medida los sombreros de paseo protegen contra la radiación UV más que las gorras. Cabe resaltar que el sombrero nunca protegerá el 100% por lo que su uso debe complementarse con otras medidas preventivas. Considerando el incremento de la radiación solar ^8^, implementar estrategias de protección, conociendo su eficacia y seguridad, es fundamental para prevenir el daño cutáneo.
